# Supplementary material for: Effect of Cellulose Nanocrystal Addition on the Physicochemical Properties of Hydroxypropyl Guar-Based Intelligent Films
Source: Membranes (Basel). 2021 Mar 29;11(4):242. doi: 10.3390/membranes11040242 (PMC8065842; doi:10.3390/membranes11040242)
Supplement: Supplementary file 1 [file membranes-11-00242-s001.pdf]

Supplementary materials

Effect of Cellulose Nanocrystal Addition on the Physicochemical Properties of Hydroxypropyl Guar-Based Intelligent Films

Yahui Meng [1,2], Yunfeng Cao [2], Kaifeng Xiong [3], Li Ma [1], Wenyuan Zhu [2], Zhu Long [4,*], Cuihua Dong [1,*]

[1] Key Laboratory of Biobased Material and Green Papermaking, Qilu University of Technology, Jinan, 250353, China. 18765382707@163.com(Y.M.); mlz.1219@163.com(L.M.)
[2] Key Laboratory of Pulp and Paper Science and Technology, Nanjing Forestry University, Nanjing, 210037, China. yunfcao@163.com(Y.C); klpp@njfu.edu.cn(W.Z)
[3] School of Light Industry and Food Engineering, Guangxi University, Nanning, 530004, China. xkf@qlu.edu.cn
[4] Key Laboratory of Eco-Textiles, Ministry of Education, Jiangnan University, Wuxi, 214122, China.
* Correspondence: longzhu@jiangnan.edu.cn(Z.L.), Tel.: +86 510 85912107; xiaodong771111@163.com(C.D.)

Table S1. Color parameters of films in different pH buffers.

pH	HPG/IL/Anth			HPG/CNC/IL/Anth			CNC/IL/Anth		
	L*	a*	b*	L*	a*	b*	L*	a*	b*
0	50.93	-1.39	13.25	62.36	6.40	12.02	59.19	11.32	0.86
2	47.84	38.41	13.83	38.13	40.31	10.13	34.49	47.27	20.96
4	49.00	14.23	6.51	46.50	23.08	5.43	39.37	35.54	4.17
6	49.11	5.66	6.15	53.00	9.14	-2.31	36.92	22.12	-4.28
8	48.92	6.54	7.01	50.11	12.79	-1.08	32.84	18.80	1.33
10	50.21	6.78	14.69	44.73	0.06	12.11	43.67	-1.11	23.61
12	54.47	-6.08	26.31	45.29	-2.05	24.15	46.99	-12.30	16.14

L* range: 100 (white) to 0 (black); a*: '+' (red) to ' - ' (green), b*: '+' (yellow) to ' - ' (blue).

Table S2. Color parameters of films in pH 12 buffer at different storage temperature.

T (℃)	HPG/IL/Anth			HPG/CNC/IL/Anth			CNC/IL/Anth		
	L*	a*	b*	L*	a*	b*	L*	a*	b*
-20	54.91	-6.10	26.97	44.02	-3.32	24.83	45.22	-11.99	16.01
-10	53.03	-6.09	26.30	43.21	-4.09	22.19	44.34	-10.05	14.06
2	54.67	-5.98	25.13	42.11	-4.15	21.04	42.55	-12.11	14.12
10	53.11	-6.91	24.61	42.49	-3.94	22.87	42.46	-10.86	13.41
50	53.23	-5.79	22.25	42.57	-4.75	22.14	43.09	-11.24	12.11
70	55.01	-4.34	20.03	49.51	-4.02	21.35	44.23	-9.71	12.60
90	56.27	-4.03	21.15	53.68	-3.16	20.36	47.48	-9.03	13.51

L* range: 100 (white) to 0 (black); a*: '+' (red) to ' - ' (green), b*: '+' (yellow) to ' - ' (blue).

Table S3. Color parameters of films at different light time.

Time (h)	HPG/IL/Anth			HPG/CNC/IL/Anth			CNC/IL/Anth		
	L*	a*	b*	L*	a*	b*	L*	a*	b*
0	50.93	-1.39	13.25	61.36	6.40	12.02	59.19	11.32	0.86
12	50.10	1.05	10.11	62.09	6.51	10.30	60.34	14.17	1.10
24	53.58	2.17	10.06	62.01	7.57	8.12	60.14	13.08	1.27
36	54.01	3.09	12.03	63.21	7.58	7.79	62.52	13.01	2.61
48	55.12	3.21	14.67	65.01	6.23	5.91	65.46	15.71	2.01
60	56.29	4.17	13.15	66.78	6.46	4.05	67.10	10.94	1.27
72	56.04	4.22	12.69	67.03	4.57	4.29	69.35	8.50	4.73

L* range: 100 (white) to 0 (black); a*: '+' (red) to ' - ' (green), b*: '+' (yellow) to ' - ' (blue).

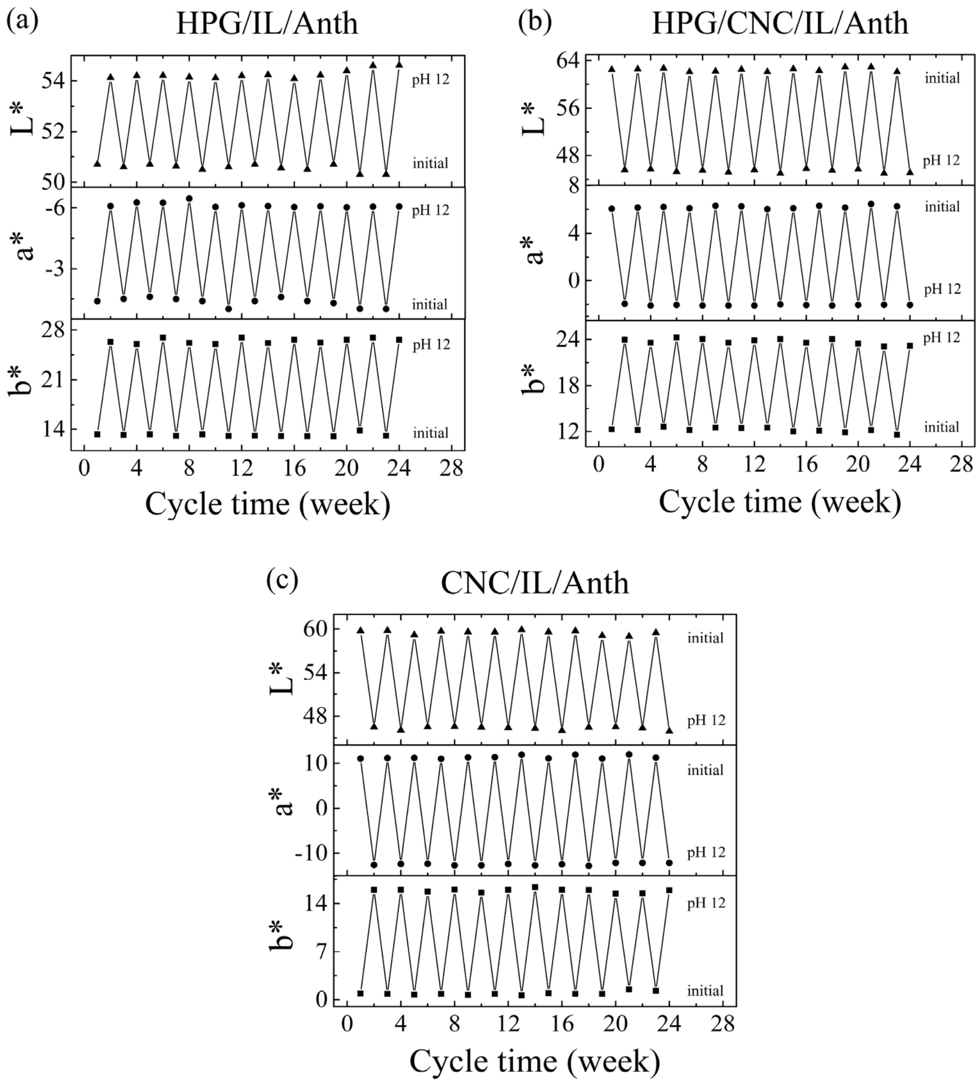

Figure S1. The color parameters L*, a*, and b*of the pH-sensing films in pH 12 buffer after long-term stored at room temperature. (a) HPG/IL/Anth, (b) HPG/CNC/IL/Anth and (c) CNC/IL/Anth.
